# Strikes and Gutters: Biomarkers and anthropometric measures for predicting diagnosed diabetes mellitus in adults in low- and middle-income countries

**DOI:** 10.1016/j.heliyon.2023.e19494

**Published:** 2023-08-30

**Authors:** Sally Sonia Simmons

**Affiliations:** Department of Social Policy, London School of Economics and Political Science, London, WC2A 2AE, United Kingdom

**Keywords:** Biomarkers, Anthropometric Indices, Diabetes, Low- and Middle-Income Countries

## Abstract

The management of diabetes necessitates the requirement of reliable health indices, specifically biomarkers and anthropometric measures, to detect the presence or absence of the disease. Nevertheless, limited robust empirical evidence exists regarding the optimal metrics for predicting diabetes in adults, particularly within low- and middle-income countries. This study investigates objective and subjective indices for screening diabetes in these countries. Methods: Data for this study was sourced from surveys conducted among adults (aged 18 years and above) in seventeen (17) countries. Self-reported diabetes status, fifty-four biomarkers, and twenty-six core and twenty-eight estimated anthropometric indices, including weight, waist circumference, body mass index, glycaemic triglycerides, and fasting blood glucose, were utilised to construct lasso regression models. Results: The study revealed variances in diabetes prediction outcomes across different countries. Central adiposity measures, fasting plasma glucose and glycaemic triglycerides demonstrated superior predictive capabilities for diabetes when compared to body mass index. Furthermore, fasting plasma or blood glucose, serving as a biomarker, emerged as the most accurate predictor of diabetes. Conclusions: These findings offer critical insights into both general and context-specific tools for diabetes screening. The study proposes that fasting plasma glucose and central adiposity indices should be considered as routine screening tools for diabetes, both in policy interventions and clinical practice. By identifying adults with or at higher risk of developing diabetes and implementing appropriate interventions, these screening tools possess the potential to mitigate diabetes-related complications in low- and middle-income countries.

## 1 Introduction

The prevalence of diabetes mellitus (diabetes) is rapidly increasing in low-income and middle-income countries (LMICs), which currently house approximately 80% of the 463 million diabetic adults worldwide [1]. Moreover, the burden of diabetes is paralleled with the escalating rates of obesity and hyperglycaemia, with around 90% of all diabetic adults in LMICs being either obese [2,3] or hyperglycaemic [4,5]. These widely recognised risk factors for diabetes can be detected through the use of anthropometric indices and biomarkers [[6], [7], [8], [9], [10], [11]].

Anthropometric measures are quantitative measurements of various physical characteristics and dimensions of the human body including body composition, size, shape, and proportions [12]. Anthropometric indices like body mass index (BMI), assess obesity and related diabetes in LMICs [3,[13], [14], [15]], a notable trend influenced by the nutrition and health policies of many LMICs. Firstly, the widespread utilisation of BMI as the primary indicator for screening malnutrition and diseases [[16], [17], [18], [19], [20], [21]]; and secondly, the focus on addressing malnutrition as a condition that primarily affects children rather than adults [16,[22], [23], [24], [25], [26]]. Consequently, there remains a significant gap in knowledge regarding the relationship between diabetes and other indices across LMICs, as well as whether the observed variations within individual countries extend to broader geographic scales in the highly heterogeneous region, LMICs [27,28]. The lack of sufficient documented evidence regarding the effectiveness of these indices in predicting diabetes in LMICs poses a barrier to the implementation of diabetes screening strategies with improved specificity and sensitivity [29]. Additionally, this limitation hinders the availability of information necessary for reforming nutrition and health guidelines and designing evidence-based, cost-effective policies to address the current diabetes epidemiology in this context. Fasting blood or plasma glucose (FPG or FBG), measured after an overnight fast, and glycosylated haemoglobin (HbA1c) have emerged as significant biomarkers in the field of diabetes screening and management [30,31]. Elevated levels of fasting glucose indicate impaired glucose regulation and may serve as an early indicator of insulin resistance and the onset of diabetes [31,32]. However, the degree to which these biomarkers, measurable indicators or substances that can be found in bodily fluids, demonstrate efficacy compared to numerous other indices for detecting diabetes in LMICs remains uncertain.

The discourse on biomarkers, anthropometric indices, and diabetes is largely confined to high-income countries (HICs), with limited attention paid to LMICs [[33], [34], [35]]. However, there have been exceptions in the form of studies that specifically concentrate on the aging Asian population and other contextual factors within LMICs [3,36,37]. Within HICs, numerous studies [5,38,39] have contributed to our understanding of diabetes prediction using various anthropometric indices and biomarkers, such as waist circumference (WC), waist-to-height ratio (WHtR), and FPG or FBG. The abundance of biomarkers and anthropometric indices in HICs supports the adoption of more accurate metrics as routine indicators for predicting diabetes [39,40]. This reduces the likelihood of misdetection in both patients and non-patients within HICs [40]. Consequently, the absence of valid and reliable evidence regarding anthropometric indices and biomarker predictors of diabetes in LMICs raises the question of whether diverse yet comparable countries require distinct or identical metrics for diabetes screening or face a high prevalence of diabetes misdetection. These uncertainties hinder the planning of targeted diabetes prevention programmes for the elderly and impede efforts to strike a balance between health, disease, and the cost of screening. Addressing these challenges would alleviate the strain on healthcare systems and contribute to achieving Sustainable Development Goal 3.4, which aims to reduce premature mortality from non-communicable diseases (NCDs) by one-third by 2030. Consequently, it is essential to emphasize the need for objective and subjective indices for predicting diabetes to address these limitations in LMICs.

The primary objective of this study is to explore the relationship between biomarkers and anthropometric measures in predicting diabetes among adults in LMICs. This research aims to address four key objectives. Firstly, the study aims to determine which specific biomarkers and anthropometric indices yield reliable outcomes for diagnosing diabetes. Secondly, the research aims to compare the predictive capabilities of biomarkers against anthropometric indices in the context of diabetes. Specifically, the study will shed light on whether biomarkers offer superior predictive accuracy compared to anthropometric indices. Thirdly, the study seeks to examine whether there is convergence or divergence in the prediction and misdetection of diabetes across different countries.

## 2 Materials and methods

### 2.1 Data sources and context

The study used data from four primary sources: World Health Organisation (WHO) global ageing and adult health (SAGE) wave 1 and 2 (individual surveys) (2007–2014); WHO STEPwise approach to non-communicable diseases (NCDs) risk factor surveillance (STEPS) wave 1 and 2 (2012); the Longitudinal ageing study in India (LASI) wave 1 (2017–2019); the Indonesia family life survey (IFLS) wave 5. SAGE is a longitudinal study of persons aged 18 and above selected from six LMICs, including Ghana, South Africa, and Mexico. STEPS is also a longitudinal study of key NCDs risk factors in more than 60 LMICs [41]. LASI provides information on the health and social aspects of ageing among adults across all the states and union territories in India [42,43]. Finally, the IFLS is a longitudinal study focusing on demography and health in Indonesia [44]. Generally, the surveys are nationally representative household and person-specific surveys focused on key demographic and health indicators used for population health and nutritional programme surveillance. Also, these four data sources were conducted according to legal standards for population-based studies in the respective countries.

**Fig. 1:**
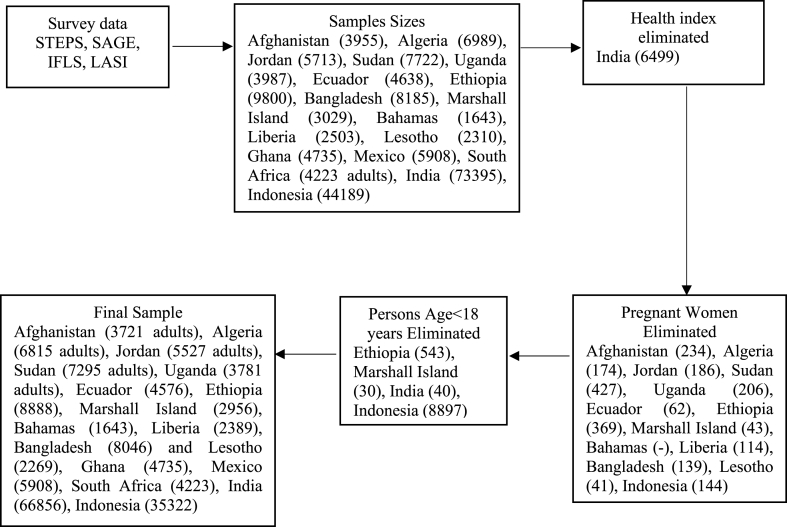
Flowchart summarizing the inclusion and exclusion criteria of the study. Source: Author's Construct based on data from Bloom et al., and [41], Kowal et al., and IFLS [[42], [43], [44],^52^].

In this study, SAGE served as a data source for three countries: Ghana, Mexico, and South Africa. Using multistage cluster sampling techniques, SAGE provides information for a representative sample of Ghanaians (4735), Mexicans (5908) and South Africans (4223); these samples were the initial participants of the study (see Table 1). In addition, data for twelve (12) countries were retrieved from the STEPS, a survey emulating the SAGE surveillance techniques. These countries were Afghanistan (3955), Algeria (6989), Jordan (5713), Sudan (7722), Uganda (3987), Ecuador (4638), Ethiopia (9800), Bangladesh (8185), Marshall Island (3029), Bahamas (1643), Liberia (2503), and Lesotho (2310). Furthermore, LASI and IFLS, provide data referent to 73396 Indians and 448139 Indonesians, respectively (see Fig. 1). Kowal and colleagues and WHO [[45], [46], [47]] provide a detailed description of the SAGE and STEPS methods of data collection and materials.

**Table 1:** Formulas for derived anthropometric indices and biomarkers.

Derived Index	Formular
Anthropometric Indices	
ABSI (body shape index)	$\frac{WC\;\left(m\right)}{{BMI}^{\frac{2}{3}}*\sqrt{H\left(m\right)}}$
AVI (abdominal volume index)	$\frac{{2*WC}^{2}\left(cm\right)+{0.7\left(WC-HC\right)}^{2}\left(cm\right)}{1000}$
BAI (body adiposity index)	$\frac{HC\left(cm\right)}{H^{1.5}\left(m\right)}-18$
BF% (body fat percentage) _male_	[1.20*BMI]+0.23*age]−16.2
BF%_female_	[1.20*BMI]+0.23*age]−5.4
BMI (body mass index)	$\frac{W\;\left(kg\right)}{H\left(m^{2}\right)}$
BRI (body roundness index)	$364.2-365.5*\sqrt{1-(\frac{{(WC\left(m\right)/2\pi )}^{2}}{{\left(0.5*H\left(m\right)\right)}^{2}}})$
BI (broca Index)	H(cm)−100
CI (conicity index)	$\frac{WC\left(m\right)}{\sqrt[{0.109}]{\frac{W\left(kg\right)}{H\left(m\right)}}}$
FFM (fat free mass)	$W\left(kg\right)*\left(1-\left(\frac{BF\%}{100}\right)\right)$
FFMI (fat free mass index)	$\frac{FFM}{H\left(m^{2}\right)}$
HI (hip index)	$\frac{HC*\;W\left(kg\right)}{{average\left(W\right)}^{0.482}}*\frac{H\;\left(cm\right)}{{average\;\left(H\right)}^{0.310}}$
LBM (lean body mass)_female_	(0.29569×W(kg))+(0.41813×H(cm))−43.2933
LBM_male_	(0.32810×W(kg))+(0.33929×H(cm))−29.5336
PI or RI (ponderal index or rohrer's index)	$\frac{W\left(kg\right)}{H\left(m^{3}\right)}$
RPI (reciprocal-ponderal index)	$\frac{H\left(m\right)}{{W\left(kg\right)}^{1/3}}$
RFM (relative fat mass) _female_	$76-20*\frac{H\;\left(m\right)}{WC\;\left(cm\right)}$
RFM_male_	$64-20*\frac{H\;\left(m\right)}{WC\;\left(cm\right)}$
VAI (visceral adiposity index)_female_	$\frac{WC\;\left(cm\right)}{36.58+\left(1.89*BMI\right)}*\frac{TG\left(mg/L\right)}{0.81}*\frac{1.52}{HDL\left(mg/L\right)}$
VAI_male_	$\frac{WC\;\left(cm\right)}{39.68+\left(1.88*BMI\right)}*\frac{TG\left(mg/L\right)}{1.03}*\frac{1.31}{HDL\left(mg/L\right)}$
WHR (waist-to-hip ratio)	$\frac{WC\;\left(cm\right)}{HC\;\left(cm\right)}$
WHT.5R (waist-to-height^0.5 ratio)	$\frac{WC}{H^{0.5}}$
WHtR (waist-to-height ratio)	$\frac{WC\;\left(m\right)}{H\left(m^{2}\right)}$
WWI (weighted-waist index)	$\frac{WC\;\left(cm\right)}{\sqrt{W\;\left(kg\right)}}$
Biomarkers	
AC (atherogenic coefficient)	$\frac{TC\left(mg/dl\right)-HDL\left(mg/dl\right)}{HDL\left(mg/dl\right)}$
AIP (atherogenic index of plasma)	$\log\left[\frac{TG\left(mg/dl\right)}{HDL\left(mg/dl\right)}\right]$
CR or CRR or CRI I (cholesterol ratio or cardiac risk ratio or castell's risk index-I)	$\frac{TC\left(mg/dl\right)}{HDL\left(mg/dl\right)}$
CRI -II (CRI II (castelli's risk index-II) or LDL-HDL ratio (low-density lipoprotein cholesterol- high-density lipoprotein cholesterol)	$\frac{LDL\left(mg/dl\right)}{HDL\left(mg/dl\right)}$
LDL (low-density lipoprotein cholesterol)	TC(mg/dl)−(HDL(mg/dl)+0.38*TG(mg/dl)
LAP (lipid accumulation product)_female_	[WC(cm)−58]*[TG(mmol/l)]
LAP _male_	[WC(cm)−65]*[TG(mmol/l)]
THR (triglyceride-high-density lipoprotein cholesterol ratio)	$\frac{TG\left(mg/dl\right)}{HDL\left(mg/dl\right)}$
TyG (triglycerides glucose index)	$\log\left[\frac{TG\left(mg/dl\right)*FBG\;or\;FPG\left(mg/dl\right)}{2}\right]$
VLDL (very low-density lipoproteins)	$\frac{TG\left(mg/dl\right)}{5}$

Source: Adapted from Amato et al., [6]; Bhowmik et al., [48]; Christakoudi et al., [49]; Gimeno‐Orna et al., [50]; WHO [51]. Note: abdominal volume index (AVI), atherogenic coefficient (AC), atherogenic index of plasma (AIP), body adiposity index (BAI), body fat percentage (BF%), body mass index (BMI), body roundness index (BRI), body shape index (ABSI), Broca index (BI), cardiac risk ratio (CRR), Castelli's risk index-I (CRI- I), Castelli's risk index-II (CRI -II), cholesterol ratio (CR), conicity index (CI), fasting blood glucose or fasting plasma glucose (FBG or FPG), fat free mass (FFM), fat free mass index (FFMI), height (H), hip circumference (HC), hip index (HI), high-density lipoprotein cholesterol (HDL), lean body mass (LBM), lipid accumulation product (LAP), low-density lipoprotein cholesterol (LDL), ponderal index (PI or RI), reciprocal ponderal index (RPI), relative fat mass (RFM), Rohrer's index or corpulence index, total cholesterol (TC), triglycerides (TG), triglycerides glucose index (TyG), triglycerides HDL ratio (THR), very low-density lipoproteins (VLDL), waist circumference (WC), waist to height ratio (WHtR), waist to height.5 ratio (WHtR), waist to hip ratio (WHR), weight (W), and weight-adjusted waist index (WWI).

A comprehensive multi-country study provides insights into patterns, similarities, and differences among the countries of interest, which can inform policy implementation, serve as a basis for analytical analyses, and enable the prediction of trends in other countries [53]. Cross-country health comparative studies involving these nations present unique and challenging epidemiological characteristics [[54], [55], [56]]. For instance, the Marshall Islands rank among the top five most obese and overweight countries globally [57]. Meanwhile, India has the world's second-largest diabetic population and the fastest-growing obesity rate [58,59]. Bangladesh and Afghanistan, which share historical, cultural, linguistic, and heritage ties with India, face similar public health challenges [60,61]. Additionally, the Global Nutrition Report [62,63] highlights that Algeria and Jordan have obesity rates higher than the regional average for the Middle East and North Africa (MENA) region. In contrast, Sudan, a North African country, exhibits lower obesity rates than the regional average, resulting in a minimal number of cardiometabolic disease-related deaths [64]. But religious practices and dietary habits in countries like Afghanistan, Algeria, Sudan, and Jordan [65] are associated with hypotension, hypoglycaemia, sleeplessness, and severe migraines [66], which are risk factors for diabetes [67]. Prolonged political unrest, malnutrition, and inadequate healthcare services significantly impact countries such as Afghanistan, Sudan, Uganda, Ethiopia, and Liberia [[68], [69], [70]] and resulting in increased incidents of disease detection errors and misdiagnosis[71,72]. Genetic predisposition and HIV-related conditions also contribute to the burden of cardiometabolic diseases, including diabetes, in countries such as Mexico, South Africa, and Lesotho [27,58,59,[73], [74], [75], [76], [77], [78], [79], [80], [81]]. Therefore, comprehending the convergence and divergence of health metric outcomes among these 17 countries entails a complex variable-oriented approach to analysing cases of diabetes in LMICs [53].

### 2.2 Measurements

The study incorporated variables from five domains: sociodemographic (such as age, sex, ethnicity or race, and caste), diseases (specifically diabetes), health (including pregnancy), biomarkers and anthropometrics (such as height, weight, waist circumference [WC], hip circumference [HC], grip strength, pulse or heart rate, blood pressure [systolic and diastolic], cholesterol [total, triglycerides], blood glucose, and haemoglobin [Hb]), and geography (consisting of country names). These variables provide essential information on cross-national factors that influence and reflect the health status of LMICs at both macro- and micro-levels. By incorporating these variables, the study aims to develop comprehensive, precise, complete, and accurate models to address the research questions effectively [82,83].

#### 2.2.1 Dependent variable

The primary dependent variable in this study is a binary indicator of self-reported diabetes. It is defined as "yes" if the respondent has been diagnosed with or informed by a doctor or health professional that they have diabetes, and "no" if the respondent does not have diabetes. The responses to the question, "Have you ever been diagnosed with or told by a doctor or health professional that you have diabetes?" were used to determine the binary indicators. Diabetes was chosen as the dependent variable due to the well-established relationship between biomarkers, anthropometric indices, and the presence of diabetes [83,84].

#### 2.2.2 Independent variable

A total of fifty-four (54) biomarkers and anthropometric indices were utilised as predictor metrics in this study. These indices have been shown to have significant associations with cardiometabolic diseases, including diabetes, at various concentrations [34,84]. The initial set consisted of twenty-six (26) measured anthropometric indices and biomarkers, such as weight, height, hip circumference (HC), waist circumference (WC), fasting plasma glucose (FBG or FPG), and triglycerides (TG). Additionally, twenty-eight (28) derived biomarkers and anthropometric indices were computed based on recommended estimations by the World Health Organisation (WHO) [10,11]. To ensure uniformity and meaningful computations, all biomarkers were transformed from mmol/l to mg/dl. However, triglycerides (TG) were transformed from mg/dl to mmol/l for the estimation of lipid accumulation product (LAP) [29] (refer to Table 1 for details). It is worth noting that the SAGE and LASI cohorts had fewer available biomarker data compared to the STEPs cohort. For specific information regarding the number of indices used as predictors in each country, please refer to Table 5 in the appendix.

#### 2.2.3 Covariate

The study incorporated a set of sociodemographic characteristics as control variables. These variables included age, sex, race/ethnicity, and caste. Age was measured in completed years, while sex was categorised as male or female. The variable for race encompassed various ethnic or racial identities, and caste represented the respondents' self-identification into specific social groups within the caste system. These sociodemographic variables were included as control variables to account for their potential influence on the relationship between the predictor variables (biomarkers and anthropometric indices) and the dependent variable (diabetes). By controlling for these sociodemographic characteristics, the study aimed to isolate and assess the specific effects of the biomarkers and anthropometric indices on diabetes outcomes. Also, this helped to ensure that any observed differences in the countries are not due to confounding factors.

#### 2.2.4 Elimination criteria, transformations, imputations and checks

The study implemented certain exclusion criteria, including pregnancy status, age, and the presence of at least one biomarker or anthropometric index. Pregnancy status was a binary measure, with a value of "1″ indicating that the respondent was pregnant and "2″ indicating that they were not. Consequently, only non-pregnant females and individuals aged 18 years or older were included in the analysis (refer to Fig. 1). This exclusion was necessary because pregnant females undergo rapid changes in body shape, size, fat accumulation, and biochemistry due to their medical condition. Including pregnant females in the sample could potentially introduce confounding factors and weaken the statistical analyses [85,86]. Additionally, though the age of majority in LMICs varies across countries [87], individuals generally gain legal autonomy over their decisions and actions related to their health from the age of 18 [88]. Therefore, the study focused on respondents aged 18 years or older. Additionally, respondents who lacked information on biomarkers and anthropometric indices were excluded from the analysis (refer to Fig. 1). Imputation methods were employed to address missing values for covariates. This approach aimed to prevent a reduction in precision and mitigate potential biases in parameter estimates [89].

To address the issue of variable dominance and potential biases, all independent variables (biomarkers and anthropometric indices) were rescaled to a common scale between 0 and 1. This enhanced a balanced consideration of variables with different value ranges. To ensure robust and generalisable predictive models, the data for each country was split into train and test sets. The train set (80% of the data) was used for model training and parameter selection, while the test set (20% of the data) was used to evaluate model performance and prevent overfitting. A high-class imbalance was identified during the preliminary analysis, where the majority of responses (73%–98%) were classified as "no" for diabetes, while the minority (1.1%–27%) were classified as "yes." This class imbalance can lead to biased predictions and reduced model performance. To address this issue and prevent classification errors, oversampling techniques were employed. Specifically, the minority class ("yes" responses indicating diabetes) was oversampled, increasing its representation in the training data. This helps alleviate the class imbalance and improves the model's ability to accurately predict both classes [90].

### 2.3 Statistical analysis

#### 2.3.1 Descriptive

Descriptive statistics were utilised to examine the distribution of variables within each country. Categorical variables, such as sex, race/ethnicity, and diabetes status, were presented as percentages (%), indicating the proportion of respondents falling into each category. Numerical variables, including age, biomarkers, and anthropometric indices, were reported as means and standard deviations (SD), providing information about the central tendency and variability of the data. By presenting these descriptive statistics, the study aimed to provide a comprehensive overview of the sample characteristics and the distribution of key variables within each country. This information allows for a better understanding of the demographic and health-related characteristics of the study population and serves as a foundation for subsequent analyses and interpretation of the findings.

#### 2.3.2 Inferential

The study employed the least absolute shrinkage and selection operator (lasso) method to identify the key biomarkers and anthropometric indices that predict diabetes in LMICs. This approach extends the lasso regularisation to a generalised linear model, allowing for the incorporation of a categorical indicator (diabetes) through a link function. The lasso algorithm is particularly useful in the presence of multicollinearity [91] as it detects patterns in the data while avoiding overfitting by selecting the most relevant predictors from a larger set of features [92,93]. Specifically, the lasso model shrinks some coefficients towards zero by imposing penalties and carries out parameter estimations, effectively filtering out less correlated and unnecessary covariates [94]. This feature makes the lasso method advantageous compared to ridge regression [95]. The appropriate adjustment parameter for the lasso model was determined using cross-validation, a process that optimizes prediction performance while producing parsimonious outcomes [96]. The lasso model has been widely employed in previous studies on disease epidemic prediction, demonstrating its efficiency and effectiveness in guiding control policies for various diseases [97,98].

The training data was used to fit the lasso model for each country, while the test sets were utilised to evaluate the performance of the models. After controlling for age, race/ethnicity, caste, and sex, biomarkers and anthropometric indicators with nonzero coefficients were identified and reported as predictors of diabetes. A subset of all datasets were selected and analysed using the same analyses performed on the overall dataset, evaluating the predictive power of anthropometric measures and biomarkers in relation to diabetes. This allowed for an assessment of the similarity between the countries and subset data.Various performance metrics were calculated to assess the models' performance. These included accuracy, sensitivity, specificity, and false positive and false negative rates. These metrics provided valuable insights into the predictive power of the models and their ability to accurately identify individuals with diabetes. By examining these performance features, the study gained a comprehensive understanding of how well the selected biomarkers and anthropometric indices predicted diabetes in the LMICs under investigation. Also, the performance assessments help determine the models' effectiveness in correctly classifying individuals with diabetes and individuals without diabetes, providing insights into their diagnostic accuracy and reliability. Accuracy defined the ability of all models, after generating the best predictors, to correctly assign outcomes reminiscent of the information in the data. Sensitivity measured the proportion of true positive cases correctly identified by the model, while specificity measured the proportion of true negative cases correctly identified by the model. Sensitivity and specificity are important indicators of the models' performance in correctly detecting individuals with self-reported diabetes (true positives [TP]) and individuals without diabetes (true negatives [TN]) based on the information available in the data [99]. Furthermore, the magnitude of diabetes errors associated with the models was assessed as false positive and false negative. While a false positive indicated indices predicting the presence of diabetes or identifying a person as diabetic though not diabetic, a false negative indicated the absence diabetes though the person is diabetic. The study's analyses were weighted and computed with R statistical software version 4.12 [100]. R programme is a software environment and programming language designed for statistical analysis, graphical representation, and data reporting. The present study used the dplyr, mice and caret R packages for the analysis [93,[100], [101], [102]].

## 3 Results

Table 2 presents data referent to the baseline characteristics of the study. Generally, there were variances in the distribution of the baseline characteristics in LMICs. As shown in Table 2, the mean age of the respondents ranged from about 35.8 ± 13.2 in Uganda to 62.8 ± 10.6 in Mexico. Except for Afghanistan, where more (1995 [54%]) males were represented, there were many females represented in most countries, especially in Lesotho, where 66% ([1495]) of respondents were female. The prevalence of self-reported diabetes was high (27%, 17%, 13%, 12.1%) in Afghanistan, Mexico, Algeria, the Marshall Islands, and India and low in Ethiopia (98.9%), respectively. Also, the average BMI was highest (29.7 ± 6.98) in the Marshall Islands and lowest (22.9 ± 4.46) in Uganda. Moreover, average BPd and BPs were largest (85.9 ± 13.4; 131 ± 21) in Lesotho and smallest (74.2 ± 4.6; 104 ± 11.8) in Mexico. The mean of central adiposity indices like WHR and BF% were highest and lowest in Mexico and the Marshall Islands, and Ethiopia, respectively. The average FPG was highest in the Marshall Islands and lowest in Ethiopia. The prevalence of FBG was high in Algeria and Bangladesh, though relatively lower than the rate in the Marshall Islands (see Table 2).

**Table 2:** Descriptive statistics of adults in LMICs.

				STEPS					IFLS								SAGE						LASI								
Predictors	Afg	Alg		Bah	Ban	Ecu	Eth		Jor	Les		Lib			Mars		Sud		Uga		Indo		Gha		Mex		SA			Ind	
Demographics and Health (Mean ± SD or N [%]) rowhead																															
Population	3721 (100%)	6815 (100%)		1643 (100%)	8046 (100%)	4576 (100%)	8888 (100%)		5527 (100%)	2269 (100%)		2389 (100%)		2956 (100%)			7295 (100%)		3781 (100%)		35322 (100%)		4735 (100%)		5908 (100%)			4223 (100%)		66856 (100%)	
Age	37.8 ± 14.5	41.4 ± 13.2		41.8 ± 10.8	39.3 ± 12.3	41.1 ± 14.3	36 ± 12.8		39.8 ± 13.9	43.1 ± 12.0		38.7 ± 10.6		39.8 ± 13.9			38.5 ± 13.6		35.8 ± 13.2		40.5 ± 15.5		57.1 ± 16.3		62.8 ± 10.6			60.3 ± 11.3		57.8 ± 11.6	
Sex rowhead																															
Males	1995 (53.6%)	3081 (45.2%)		645 (39.3%)	4242 (52.7%)	1944 (42.5%)	3758 (42.3%)		2203 (39.8%)	774 (34.1%)		1065 (44.6%)		1423 (48.1%)			2707 (37.1%)		1603 (42.4%)		17037 (48.2%)		1948 (41.1%)		2292 (38.8%)			1798 (42.6%)		28218 (42.2%)	
Females	1726 (46.4%)	3734 (54.8%)		998 (60.7%)	3804 (47.3%)	2632 (57.5%)	5130 (57.7%)		3324 (60.2%)	1495 (65.9%)		1324 (55.4%)		1533 (51.9%)			4588 (62.9%)		2178 (57.6%)		18285 (51.8%)		2787 (58.9%)		3616 (61.2%)			2425 (57.4%)		38638 (57.8%)	
Race/Ethnicity rowhead																															
Asian	NA	NA		NA	NA	NA	NA		NA	NA		NA		NA			NA		NA		NA		NA		NA			382 (9.1%)		NA	
Black	NA	NA		NA	NA	154 (3.4%)	NA		NA	NA		NA		NA			NA		NA		NA		NA		NA			2696 (63.8%)		NA	
Indigenous	NA	NA		NA	NA	370 (8.1%)	NA		NA	NA		NA		NA			NA		NA		NA		NA		NA			NA		NA	
Mestizo						3854 (84.2%)	NA		NA	NA		NA		NA			NA		NA		NA		NA		NA			NA		NA	
Mixed	NA	NA		NA	NA	71 (6.1%)	NA		NA	NA		NA		NA			NA		NA		NA		NA		NA			813 (19.3%)		NA	
White	NA	NA		NA	NA	119 (2.6%)	NA		NA	NA		NA		NA			NA		NA		NA		NA		NA			324 (7.7%)		NA	
Other						8 (0.2%)	NA		NA	NA		NA		NA			NA		NA		NA		NA		NA			8 (0.19%)		NA	
Caste rowhead																															
Yes	NA	NA		NA	NA	NA	NA		NA	NA		NA		NA			NA		NA		NA		NA		NA			NA		64384 (96.3%)	
No	NA	NA		NA	NA	NA	NA		NA	NA		NA		NA			NA		NA		NA		NA		NA			NA		2472 (3.6%)	
Diabetes rowhead																															
Yes	998 (27%)	916 (13%)		161 (10%)	536 (6.7%)	291 (6.4%)	97 (1.1%)		662 (12%)	62 (2.7%)		30 (1.3%)		362 (12.1%)			391 (5.4%)		44 (1.2%)		778 (2.2%)		87 (1.8%)		986 (16.7%)			370 (8.8%)		8060 (12.1%)	
No	2723(73%)	5899 (87%)		1482 (90%)	7510 (93.3%)	4285 (93.6%)	8791 (98.9%)		4865 (88%)	2207 (97.3%)		2359 (98.7%)		2594 (87.8%)			6904 (94.6%)		3737 (98.8%)		34544 (97.8%)		4648 (98.2%)		4922 (83.3%)			3653 (91.2%)		58796 (87.9%)	
Anthropometric Indices (Mean ± SD) rowhead																															
ABSI	0.13 ± 0.02	0.13 ± 0.6	0.11 ± 0.2		0.13 ± 0.01	0.13 ± 0.01		0.12 ± 0.01	0.12 ± 0.01		0.12 ± 0.4		0.11 ± 0.05			NA		0.13 ± 0.02		0.12 ± 0.05		0.13 ± 0.01		0.13 ± 0.02		0.14 ± 0.01			0.12 ± 0.03		0.14 ± 0.01
Arm C	NA	NA	NA		NA	NA		NA	NA		NA		NA			30.3 ± 4.34		NA		NA		NA		NA		NA			NA		NA
AVI	NA	18.2 ± 6.1	NA		13.2 ± 3.6	NA		12 ± 3.6	18.5 ± 18.0		15.6 ± 14.7		13.0 ± 6.5			NA		15.2 ± 7.1		13 ± 4.2		14.6 ± 4.1		13.7 ± 3.7		16.9 ± 2.7			14.5 ± 4.8		15.0 ± 4.4
BAI	NA	30.9 ± 6.8	NA		29 ± 6.3	NA		26.5 ± 5.9	32.2 ± 8.5		32 ± 8.5		28.8 ± 11.5			NA		NA		28.3 ± 12.1		30.3 ± 6.4		28.1 ± 12.1		33 ± 3.2			32.5 ± 12.4		29.1 ± 5.8
BF%	27.9 ± 10.0	31.6 ± 10.4	32.7 ± 11.3		27.7 ± 7.8	32.3 ± 9.6		23.4 ± 7.7	34 ± 11.9		32.5 ± 11.8		31.5 ± 14.0			35.4 ± 12.6		28.0 ± 10.2		25.8 ± 9.0		27.0 ± 9.1		28.6 ± 6.9		31.1 ± 5.4			31.6 ± 5.2		30.9 ± 7.9
BI	61.9 ± 10.1	65.8 ± 9.9	66.6 ± 9.7		56.6 ± 9.1	58.2 ± 9.4		62.6 ± 9.05	63.9 ± 9.7		59.6 ± 9.7		57.3 ± 13.5			57.9 ± 10.1		62.3 ± 18.6		61.8 ± 9.0		56.4 ± 8.5		57.9 ± 9.7		55.4 ± 9.5			58.4 ± 11.0		55.3 ± 8.8
BMI	25.3 ± 5.9	26.9 ± 5.5	28.6 ± 7.2		23.1 ± 4.4	27.4 ± 5.0		22.9 ± 3.5	28.7 ± 6.6		26.2 ± 6.8		26.5 ± 6.7			29.7 ± 7.0		23.7 ± 5.7		22.9 ± 4.5		23.5 ± 4.5		23.9 ± 5.3		26.8 ± 5.4			27.7 ± 8.1		23.1 ± 4.6
BPd	81.1 ± 12.0	76.4 ± 10.7	82.6 ± 11.8		80 ± 12.1	76.4 ± 10.7		79.2 ± 11.8	79.6 ± 10.6		85.9 ± 13.4		80.0 ± 13.5			75.3 ± 12.9		84.9 ± 11.0		82.7 ± 12		79.1 ± 11.2		78.2 ± 12.9		74.2 ± 4.6			75.1 ± 8.5		81.7 ± 10.3
BPs	125 ± 15.2	126 ± 17.9	128 ± 17.0		122 ± 17.7	121 ± 16.1		122 ± 17.5	119 ± 17.3		131 ± 21		128 ± 20			121 ± 18.7		130 ± 18.1		127 ± 17.6		126 ± 15.8		128 ± 21.6		104 ± 11.8			107 ± 13.7		128 ± 19
BRI	3.84 ± 0.9	4.31 ± 0.9	3.76 ± 2.1		4.85 ± 1.1	4.89 ± 0.6		4.11 ± 0.9	5.20 ± 1.4		5.04 ± 1.4		4.62 ± 2.1			NA		4.64 ± 1.2		4.39 ± 1.0		3.81 ± 1.1		3.59 ± 1.1		4.65 ± 0.9			3.88 ± 1.4		4.45 ± 1.4
CI	0.81 ± 0.2	0.88 ± 0.4	0.69 ± 0.3		0.74 ± 0.1	0.83 ± 0.1		0.7 ± 0.1	0.85 ± 0.2		0.79 ± 0.3		0.71 ± 0.3			NA		0.78 ± 0.1		0.74 ± 0.3		0.77 ± 0.1		0.78 ± 0.2		0.82 ± 0.1			0.83 ± 0.2		0.79 ± 0.1
FFM	47 ± 9.3	48. ±7.4	49.5 ± 8.1		42 ± 6.1	45 ± 7.8		42.1 ± 7.5	48.2 ± 7.2		43.7 ± 7.1		43.8 ± 11.5			45 ± 4.2		44.9 ± 8.5		43.9 ± 7.7		41.5 ± 8.2		42.9 ± 8.1		44.6 ± 8.4			43.1 ± 8.9		40 ± 8.05
FFMI	17.8 ± 2.3	17.7 ± 1.7	18.7 ± 2.2		17 ± 2.6	17.9 ± 2.1		15.8 ± 1.9	18.2 ± 2.0		17.2 ± 2.4		17.8 ± 2.5			17.9 ± 1.3		17 ± 2.2		16.7 ± 2.1		16.8 ± 1.5		17.1 ± 1.5		17.8 ± 2.1			17.1 ± 2.4		16.1 ± 1.7
GripS_L	NA	NA	NA		NA	NA		NA	NA		NA		NA			NA		NA		NA		24.6 ± 7.3		23.6 ± 10.7		22 ± 8.4			25.1 ± 5.6		20.3 ± 7.6
GripS_R	NA	NA	NA		NA	NA		NA	NA		NA		NA			NA		NA		NA		25.9 ± 7.5		25.7 ± 11.2		23.3 ± 8.6			27.7 ± 7.0		22.4 ± 8.1
HC	NA	104 ± 12.9	NA		91.6 ± 9.1	NA		89.9 ± 10.6	105 ± 14.3		99.9 ± 15.4		91.1 ± 23.3			NA		NA		93.4 ± 12.0		92.6 ± 9.98		92.4 ± 15.5		98.4 ± 6.6			96.5 ± 17.2		91.2 ± 9.9
Height	162 ± 9.5	166 ± 9.9	167 ± 9.7		157 ± 9.1	158 ± 9.4		163 ± 9.1	164 ± 9.7		160 ± 9.7		157 ± 13.5			158 ± 10.1		162 ± 18.6		162 ± 9.0		156 ± 8.5		161 ± 11.2		155 ± 9.5			158 ± 12.6		155 ± 8.9
HI	NA	106 ± 31.7	NA		92.8 ± 26.3	NA		90.8 ± 24.3	107 ± 37.7		103 ± 41.2		93 ± 40.2			NA		NA		94.8 ± 31.6		94.7 ± 28.2		93.9 ± 22.2		103 ± 19.8			99.7 ± 3		92.1 ± 20.1
HRate	NA	77.7 ± 11.4	79.7 ± 12.3		77.5 ± 11.9	75.5 ± 11.6		78 ± 12.6	78.1 ± 11.0		74.3 ± 12.4		79.7 ± 14.4			NA		81.0 ± 12.6		74.5 ± 13.6		NA		NA		NA			NA		NA
KH	NA	NA	NA		NA	NA		NA	NA		NA		NA			NA		NA		NA		47.5 ± 3.18		NA		NA			NA		NA
LBM	45.5 ± 8.0	49.0 ± 7.6	50.1 ± 8.6		41.9 ± 5.3	44.5 ± 7.9		42.1 ± 6.3	49.1 ± 8.2		44.1 ± 7.0		43.5 ± 7.7			44.3 ± 8.6		45 ± 7.4		43.2 ± 6.6		40.4 ± 6.8		43.8 ± 6.8		44 ± 7.6			43.7 ± 7.2		39.3 ± 7.2
PI or RI	15.8 ± 4.3	16.4 ± 4.2	18.5 ± 5.1		14.8 ± 3.3	17.4 ± 3.5		12.9 ± 2.5	17.7 ± 4.7		16.7 ± 5.5		17.9 ± 9.2			19.2 ± 6.4		14.6 ± 4.3		14.2 ± 3.2		15.1 ± 3.1		14.9 ± 3.6		16.6 ± 2.2			18.3 ± 4.3		16.1 ± 3.0
PRate	NA	NA	NA		NA	NA		NA	NA		NA		NA			NA		NA		NA		76.9 ± 11.1		77.1 ± 11.7		75.6 ± 4.99			77.8 ± 7.29		70.3 ± 11.4
RFM	69.2 ± 6.0	70.2 ± 6.0	70.7 ± 5.9		69.3 ± 6.0	70.6 ± 5.94		70.5 ± 5.9	70.8 ± 5.9		71.5 ± 5.7		70.2 ± 6.1			NA		71.2 ± 5.8		70.5 ± 6.0		69.9 ± 6.1		70.7 ± 5.9		71 ± 5.9			70.5 ± 5.9		70.6 ± 5.9
RPI	0.40 ± 0.03	0.40 ± 0.03	0.38 ± 0.3		0.4 ± 0.3	0.39 ± 0.3		0.42 ± 0.3	0.39 ± 0.3		0.4 ± 0.4		0.39 ± 0.4			0.38 ± 0.3		0.42 ± 0.3		0.4 ± 0.3		0.4 ± 0.3		0.4 ± 0.4		0.38 ± 0.2			0.38 ± 0.4		0.4 ± 0.2
UAL	NA	NA	NA		NA	NA		NA	NA		NA		NA			NA		NA		33.6 ± 3.3		NA		NA		NA			NA		NA
WC	83.7 ± 17.5	89.8 ± 14.7	74.9 ± 29.8		80.1 ± 11.2	90.2 ± 12.0		76.1 ± 10.2	87.9 ± 16.2		85.3 ± 13.7		76.7 ± 20.8			NA		83.8 ± 15.9		79.1 ± 9.82		84.3 ± 11.9		84.6 ± 15.8		87.2 ± 12.1			86.7 ± 17.1		85.4 ± 12.6
Weight	66.7 ± 15.2	73.1 ± 14.9	73.8 ± 19.9		57.3 ± 11.3	68.6 ± 14.2		56.3 ± 10.0	72 ± 17.7		66.7 ± 16.5		66.1 ± 15.9			74.5 ± 18.7		63.8 ± 15.7		59.9 ± 12.5		61.8 ± 15.1		63.9 ± 15.4		70.5 ± 13.8			71.9 ± 17.2		62.9 ± 10.1
WHR	NA	0.91 ± 0.1	NA		0.88 ± 0.1	NA		0.85 ± 0.1	0.89 ± 0.1		086 ± 0.1		0.85 ± 0.1			NA		NA		0.85 ± 0.1		0.91 ± 0.1		0.89 ± 0.1		0.94 ± 0.1			0.9 ± 0.2		0.93 ± 0.1
WHT.5R	6.90 ± 1.4	6.9 ± 0.7	5.82 ± 2.3		6.40 ± 0.9	7.18 ± 0.9		5.97 ± 0.8	7.23 ± 1.3		6.85 ± 2.6		6.22 ± 2.8			NA		6.63 ± 1.1		6.32 ± 2.5		6.78 ± 1.0		6.68 ± 1.3		7.2 ± 0.6			6.88 ± 1.5		6.9 ± 1.01
WHtR	0.34 ± 0.1	0.35 ± 0.2	0.37 ± 0.1		0.33 ± 0.1	0.36 ± 0.1		0.29 ± 0.1	0.35 ± 0.1		0.34 ± 0.1		0.32 ± 0.1			NA		0.32 ± 0.1		0.31 ± 0.1		0.36 ± 0.1		0.34 ± 0.1		0.41 ± 0.1			0.37 ± 0.1		0.36 ± 0.1
WWI	10.9 ± 1.8	11.2 ± 5.2	9.21 ± 3.1		10.7 ± 0.9	10.9 ± 0.9		10.3 ± 1.1	10.6 ± 1.2		10.7 ± 4.4		9.70 ± 4.6			NA		10.7 ± 1.3		10.4 ± 4.1		10.9 ± 1.0		10.7 ± 1.7		10.9 ± 1.4			10.6 ± 2.4		11.6 ± 1.0
Biomarkers (Mean ± SD) rowhead																															
A1C-DBS	NA	NA	NA		NA	NA		NA	NA		NA		NA			NA		NA		NA		5.95 ± 1.1		NA		NA			NA		NA
AC	NA	2.80 ± 1.1	NA		3.22 ± 1.0	NA		2.51 ± 1.2	3.1 ± 1.6		2.44 ± 1.4		NA			NA		2.99 ± 2.1		2.65 ± 1.5		NA		NA		NA			NA		NA
AIP	NA	0.43 ± 0.04	NA		0.52 ± 0.4	NA		0.45 ± 0.1	0.61 ± 0.2		NA		NA			NA		NA		NA		NA		NA		NA			NA		NA
CR III or LHR	NA	2.73 ± 0.7	NA		3.7 ± 1.1	NA		1.4 ± 1.3	1.84 ± 0.7		NA		NA			NA		NA		NA		NA		NA		NA			NA		NA
CR or CRII or CRR	NA	3.7 ± 1.1	NA		4.01 ± 1.4	NA		3.53 ± 1.5	3.85 ± 1.6		3.24 ± 1.4		NA			NA		4.±1.2		3.65 ± 1.5		NA		NA		NA			NA		NA
Crp-*p*-equ	NA	NA	NA		NA	NA		NA	NA		NA		NA			NA		NA		NA		2.03 ± 3.4		NA		NA			NA		NA
Crp -dbs	NA	NA	NA		NA	NA		NA	NA		NA		NA			NA		NA		NA		2.86 ± 3.8		NA		NA			NA		NA
FPG	88.5 ± 22	95.6 ± 19.1	NA		94.2 ± 17.1	90.5 ± 19.5		81.4 ± 17.0	89.0 ± 26.8		83.9 ± 46.2		93.5 ± 17.1			103 ± 29.2		93.2 ± 13.6		71 ± 0.2		NA		NA		NA			NA		NA
Hb	NA	NA	NA		NA	NA		NA	NA		NA		NA			NA		NA		NA		13.4 ± 1.88		NA		NA			NA		NA
HbA1C or A1C-REV	NA	NA	NA		NA	NA		NA	NA		NA		NA			NA		NA		NA		5.69 ± 1.14		NA		NA			NA		NA
HDL	NA	45.8 ± 12.2	NA		38.8 ± 9.8	NA		42.1 ± 13.4	32.9 ± 5.2		28.4 ± 8.3		NA			NA		22.8 ± 9.7		19.4 ± 9.7		NA		NA		NA			NA		NA
LAP	NA	101 ± 45.5	NA		154 ± 75.7	NA		128 ± 43.8	123 ± 49.7		NA		127 ± 39.8			NA		NA		NA		NA		NA		NA			NA		NA
LDL	NA	131 28.4	NA		163.1 ± 18.9	NA		125.1 ± 28.9	141 ± 15		NA		NA			NA		NA		NA		NA		NA		NA			NA		NA
Potassium	NA	NA	NA		NA	NA		NA	NA		NA		NA			NA		40.3 ± 25.5		NA		NA		NA		NA			NA		NA
TC	147 ± 37.5	150 ± 33.7	NA		153 ± 38.9	153 ± 42.2		138 ± 33.8	151.6 ± 19		104 ± 15.6		140 ± 38.8			156 ± 39.3		79.9 ± 20.2		63.1 ± 17.4		NA		NA		NA			NA		NA
TG	NA	105.4 ± 37.7	NA		107 ± 61.8	NA		117 ± 10.3	126 ± 15.7		NA		99 ± 26.4			NA		NA		NA		NA		NA		NA			NA		NA
THR	NA	2.2 ± 1.1	NA		2.86 ± 1.3	NA		2.67 ± 1.3	2.34 ± 1.1		NA		NA			NA		NA		NA		NA		NA		NA			NA		NA
TyG	NA	8.24 ± 0.5	NA		8.73 ± 0.7	NA		8.37 ± 0.6	8.83 ± 0.7		NA		8.36 ± 0.4			NA		NA		NA		NA		NA		NA			NA		NA
UC	91.1 ± 31.7	NA	NA		90.4 ± 73.7	NA		NA	93.8 ± 52.5		NA		NA			NA		90.9 ± 11.6		NA		NA		NA		NA			NA		NA
US	140 ± 75.5	NA	NA		124 ± 69.4	NA		NA	150 ± 71.7		NA		NA			NA		121 ± 61.4		NA		NA		NA		NA			NA		NA
VAI	NA	2.29 ± 0.4	NA		2.19 ± 0.2	NA		2.08 ± 0.7	2.14 ± 0.9		NA		NA			NA		NA		NA		NA		NA		NA			NA		NA
VLDL	NA	18.9 ± 2.7	NA		21.7 ± 7.09	NA		20.4 ± 2.06	20.4 ± 21.8		NA		19.9 ± 5.3			NA		NA		NA		NA		NA		NA			NA		NA

Source: Computed from Refs. [103,104]. Note: Afghanistan (Afg), Algeria (Alg), Bahamas (Bah), Ecuador (Ecu), Ethiopia (Eth), Jordan (Jor), Lesotho (Les), Liberia (Lib), Marshall Islands (Mar), Bangladesh (Ban), Sudan (Sud), Uganda (Uga), Indonesia (Indo), Ghana (Gha), Mexico (Mex), South Africa (SA), India (Ind). Number of observations are presented as frequencies (N) (percentages [%]); biomarkers and anthropometric indices are presented as mean ± standard deviation (SD); not available (NA). Categorical values are presented as frequencies (N), and weighted prevalence (%). Abdominal volume index (AVI), atherogenic coefficient (AC), dried blood spot glycated haemoglobin (A1C-DBS), atherogenic index of plasma (AIP), blood pressure systolic (BPs), blood pressure diastolic (BPd) body adiposity index (BAI), body fat percentage (BF%), body mass index (BMI), body roundness index (BRI),body shape index (ABSI), broca index (BI), cardiac risk ratio (CRR), Castelli's risk index-I (CRI- I), Castelli's risk index-II (CRI -II), cholesterol ratio (CR), C-reactive protein equation (Crp-*p*-equ), conicity index (CI), dried blood spot C-reactive protein (Crp-dbs), fasting blood glucose or fasting plasma glucose (FBG or FPG), fat free mass (FFM), fat free mass index (FFMI), grip strength left hand (GripS_L), grip strength right hand (GripS_Rheight), height (H), haemoglobin (Hb), glycated haemoglobin (HbA1C or A1C-REV), hip circumference (HC), hip index (HI), high-density lipoprotein cholesterol (HDL), lean body mass (LBM), lipid accumulation product (LAP), low-density lipoprotein cholesterol (LDL), ponderal index (PI or RI), reciprocal ponderal index (RPI), relative fat mass (RFM), Rohrer's index or corpulence index, total cholesterol (TC), triglycerides (TG), triglycerides glucose index (TyG), triglycerides-HDL ratio (THR), Upper arm length (UAL), urinary creatinine (UC), urinary sodium (US), very low-density lipoproteins (VLDL), waist circumference (WC), waist to height ratio (WHtR), waist to height.5 ratio (WHtR), waist to hip ratio (WHR), weight (W), and weight-adjusted waist index (WWI).

Table 3 displays the results of biomarkers and anthropometric indices predicting diabetes in LMICs. After adjusting for demographic factors, there were variations in the prediction outcomes for diabetes across all settings. Among the anthropometric indices, two main groups emerged as strong predictors of diabetes: blood pressure measures and central adiposity measures. These indices included BPd, BPs, BAI, ABSI, BF%, BRI, WHtR, and LBM. Similarly, glycaemia and lipidaemia metrics such as FPG, TyG, TC, and TG proved to be potent biomarkers for predicting diabetes in LMICs. For example, the prediction outcomes for FPG ranged from approximately 0.9 in Uganda to 2.71 in Jordan. BMI and height were relatively weaker predictors compared to FPG across all settings. While the Marshall Islands had higher prediction outcomes for most indices, Ecuador exhibited lower prediction outcomes. FPG-inclusive measures like TyG yielded stronger prediction outcomes in Algeria, Liberia, Jordan, Ethiopia, and Bangladesh. Additionally, country-specific indices such as Crp-*p*-equ and Crp-dbs in Indonesia showed fairly strong predictions for diabetes. These findings highlight the variability in the predictive power of different biomarkers and anthropometric indices for diabetes across LMICs. Blood pressure and central adiposity measures, as well as metrics related to glycaemia and lipidaemia, were consistently robust predictors of diabetes in most countries.

**Table 3:** Biomarkers and anthropometric indices for predicting diabetes in adults in LMICs.

	STEPS																				IFLS			SAGE						LASI	
Predictors	Afg		Alg	Bah		Ban			Ecu	Eth			Jor	Les	Lib		Mars		Sud	Uga	Indo			Gha	Mex			SA		Ind	
Anthropometric Indices rowhead																															
ABSI	1.0	0.4		0.9		0.5							0.1	0.3			NA		0.0	0.8	0.5					0.8		0.4		0.4	
ArmC	NA	NA		NA		NA			NA	NA			NA	NA	NA		0.94		NA	NA	0.08			NA		NA		NA			
AVI	NA	0.7		NA						1.6			0.8		0.6		NA			0.7				0.2		0.6		0.8		0.0	
BAI	NA			NA		0.3							1.8	0.71	0.8		NA		NA	0.4				0.7		0.5		0.8		0.0	
BF%	0.1	1.0		0.39					0.2	0.					0.0		2.0			0.7	0.1			0.4		0.7					
BI rowhead																															
BMI	0.1			0.3										0.0			1.5		0.0	0.0							0.2		0.4		
BPd	0.1	0.1							0.2				0.4	0.6		0.1	0.3		0.7			0.1		0.5			0.5		0.8		0.4
BPs				0.6		0.1			0.1	0.3			0.2			0.5			0.1	0.2		0.4					0.2		0.0		0.2
BRI	0.1			0.4												0.3	NA		0.7								0.0				0.1
CI		0.0							0.0							0.1	NA		0.0	0.2											0.0
FFM		−0.1		−0.0																											
FFMI						−0.1			−0.1	−0.1			−0.1				0.0							−0.4					−0.1		−0.1
GripS_L	NA	NA		NA		NA			NA	NA			NA	NA		NA	NA		NA	NA				−0.2					−0.0		−0.0
GripS_R	NA	NA		NA		NA			NA	NA			NA	NA		NA	NA		NA	NA		−0.0		−0.3			−0.0		−0.2		−0.0
HC	NA			NA					NA							0.2	NA		NA												
Height rowhead																															
HI	NA			NA					NA								NA		NA	0.1				0.1			0.1				
HRate	NA			0.2		0.1			0.1	0.1			0.1	0.0		0.0	NA		0.0	0.2		NA		NA			NA		NA		NA
KH	NA	NA		NA		NA			NA	NA			NA	NA		NA	NA		NA	NA				NA			NA		NA		NA
LBM		−0.5							−0.1	−0.7			−0.5				−0.2					−0.5					−0.1				
PI or RI	0.01			0.45												0.1	1.3			0.6											
PRate	NA	NA		NA		NA			NA	NA			NA	NA		NA	NA		NA	NA		0.1		0.2			0.4				0.1
RFM		0.0		0.1		0.8			0.1					0.0			NA		0.1					0.7							
RPI		0.1								0.2										0.3											
UAL	NA	NA		NA		NA			NA	NA			NA	NA		NA	NA		NA	NA				NA			NA		NA		NA
WC	0.1	0.5								0.4			0.8				NA		0.3	0.2		0.4		0.0					0.0		0.2
Weight									0.1					0.0			1.2		0.0												
WHR	NA			NA		0.1			NA	0.5			0.1			0.1	NA		NA					0.6					0.2		0.6
WHT.5R																0.1	NA					0.01									
WHtR	0.9	0.5		0.7		0.4			0.3	0.8			0.8	0.2		0.1	NA			0.6				0.1			1.0		0.6		0.7
WWI													0.1				NA			0.1				0.1							
Biomarkers rowhead																															
A1C-DBS	NA	NA			NA		NA	NA			NA	NA		NA		NA		NA	NA	NA			NA				NA		NA		NA
AC	NA	0.0			NA			NA			0.5			0.0		NA		NA		0.0		NA	NA				NA		NA		NA
AIP	NA				NA			NA			0.81	0.59		NA		NA		NA	NA	NA		NA	NA				NA		NA		NA
CRI II or LHR		0.09												NA																	
CR or CRR or CRII	NA				NA		0.0	NA			0.1	0.7		0.0		NA		NA	0.2	0.0		NA	NA				NA		NA		NA
Crp -*p*-equ	NA	NA			NA		NA	NA			NA	NA		NA		NA		NA	NA	NA		1.2	NA				NA		NA		NA
Crp -dbs	NA	NA			NA		NA	NA			NA	NA		NA		NA		NA	NA	NA		1.3	NA				NA		NA		NA
FPG	1.2	1. 9			NA		2.0	1.0			1.8	2.7		1.3		1.0		2.2	1.8	0.9		NA	NA				NA		NA		NA
Hb	NA	NA			NA		NA	NA			NA	NA		NA		NA		NA	NA	NA		−0.1	NA				NA		NA		NA
HbA1c or A1C-REV	NA	NA			NA		NA	NA			NA	NA		NA		NA		NA	NA	NA		1.7	NA				NA		NA		NA
HDL	NA	NA			NA		−0.3	NA			−0.7	−0.5				NA		NA	−1.0	−0.6		NA	NA				NA		NA		NA
LAP	NA	0.0			NA			NA						NA		0.2		NA	NA	NA		NA	NA				NA		NA		NA
LDL	NA				NA		0.0	NA			0.2	1.1		NA		NA		NA	NA	NA		NA	NA				NA		NA		NA
Potassium	NA	NA			NA		NA	NA			NA	NA		NA		NA		NA	−0.3	NA		NA	NA				NA		NA		NA
TC	0.1	0.1			NA			0.7								0.3		1.0	0.2	0.1		NA	NA				NA		NA		NA
TG	NA	0.4			NA		0.6	NA			0.7	0.9		NA		0.0		NA	NA	NA		NA	NA				NA		NA		NA
THR	NA				NA			NA				0.4		NA		NA		NA	NA	NA		NA	NA				NA		NA		NA
TyG	NA	0.2			NA		0.4	NA			0.5	1.1		NA				NA	NA	NA		NA	NA				NA		NA		NA
UC	0.35	NA			NA		0.5	NA			NA	0.3		NA		NA		NA	0.0	NA		NA	NA				NA		NA		NA
US	0.10	NA			NA		0.1	NA			NA	0.2		NA		NA		NA	0.1	NA		NA	NA				NA		NA		NA
VAI	NA	0.0			NA			NA			0.3	0.5				NA		NA	NA	NA		NA	NA				NA		NA		NA
VLDL	NA	0.1			NA		0.0	NA			0.1	0.1				0.5		NA	NA	NA		NA	NA				NA		NA		NA

Source: Computed from Refs. [103,104]. Note: Afghanistan (Afg), Algeria (Alg), Bahamas (Bah), Ecuador (Ecu), Ethiopia (Eth), Jordan (Jor), Lesotho (Les), Liberia (Lib), Marshall Islands (Mar), Bangladesh (Ban), Sudan (Sud), Uganda (Uga), Indonesia (Indo), Ghana (Gha), Mexico (Mex), South Africa (SA), India (Ind). Not available (NA). Abdominal volume index (AVI), atherogenic coefficient (AC), dried blood spot glycated haemoglobin (A1C-DBS), atherogenic index of plasma (AIP), blood pressure systolic (BPs), blood pressure diastolic (BPd) body adiposity index (BAI), body fat percentage (BF%), body mass index (BMI), body roundness index (BRI),body shape index (ABSI), broca index (BI), cardiac risk ratio (CRR), Castelli's risk index-I (CRI- I), Castelli's risk index-II (CRI -II), cholesterol ratio (CR), C-reactive protein equation (Crp-*p*-equ), conicity index (CI), dried blood spot C-reactive protein (Crp-dbs), fasting blood glucose or fasting plasma glucose (FBG or FPG), fat free mass (FFM), fat free mass index (FFMI), grip strength left hand (GripS_L), grip strength right hand (GripS_Rheight), height (H), haemoglobin (Hb), glycated haemoglobin (HbA1C or A1C-REV), hip circumference (HC), hip index (HI), high-density lipoprotein cholesterol (HDL), lean body mass (LBM), lipid accumulation product (LAP), low-density lipoprotein cholesterol (LDL), rohrer's index or corpulence index, ponderal index (PI or RI), reciprocal ponderal index (RPI), relative fat mass (RFM), Rohrer's index or corpulence index, total cholesterol (TC), triglycerides (TG), triglycerides glucose index (TyG), triglycerides-HDL ratio (THR), Upper arm length (UAL), urinary creatinine (UC), urinary sodium (US), very low-density lipoproteins (VLDL), waist circumference (WC), waist to height ratio (WHtR), waist to height.5 ratio (WHtR), waist to hip ratio (WHR), weight (W), and weight-adjusted waist index (WWI).

Table 4 presents the outcomes of the models' diabetes detection performances for all countries. There were variations at the country level in terms of accuracy, sensitivity, specificity, and miss-rate (false positives and negatives). The model's performance outcomes were higher in Afghanistan, Algeria, Ecuador, Ethiopia, Jordan, Lesotho, Liberia, the Marshall Islands, Bangladesh, Sudan, Uganda, and Indonesia, while they were lower in the Bahamas, Ghana, India, Mexico, and South Africa. The accuracy, which represents the number of correct diabetes predictions relative to the data, ranged from approximately 0.74 in the Bahamas to 0.89 in Ethiopia. The true positives or sensitivity (TP) was highest in Ethiopia at 0.87 and lowest in the Bahamas at 0.78. The true negatives or specificity (TN) ranged from about 0.72 in the Bahamas to 0.93 in Indonesia. The rate of incorrectly predicted diabetic cases (false positive [FP]) was highest in the Bahamas at 0.28 and lowest in Ethiopia at 0.07. The false negatives (FN), which indicate incorrectly predicted non-diabetic cases, were highest in the Bahamas at 0.22 and lowest in Ethiopia at 0.13. These results highlight the country-level variances in the performance of the models for diabetes detection. The accuracy, sensitivity, specificity, and miss-rate outcomes varied across different countries, demonstrating the importance of considering the specific context and characteristics of each country when developing and evaluating predictive models for diabetes detection.

**Table 4:** Diagnostic performance of diabetes prediction models in LMICs.

	STEPS												IFLS	SAGE			LASI
	Afg	Alg	Bah	Ecu	Eth	Jor	Les	Lib	Mars	Ban	Sud	Uga	Indo	Gha	Mex	SA	Ind
ACC	0.81	0.84	0.74	0.85	0.89	0.88	0.83	0.84	0.80	0.86	0.84	0.83	0.87	0.75	0.77	0.76	0.76
TP	0.85	0.86	0.78	0.81	0.87	0.86	0.82	0.85	0.80	0.83	0.85	0.85	0.84	0.83	0.79	0.84	0.78
TN	0.8	0.80	0.72	0.87	0.93	0.92	0.89	0.81	0.75	0.91	0.90	0.90	0.93	0.77	0.82	0.77	0.83
FP	0.19	0.20	0.28	0.13	0.07	0.08	0.11	0.19	0.25	0.10	0.10	0.10	0.07	0.23	0.18	0.23	0.18
FN	0.15	0.14	0.22	0.19	0.13	0.14	0.18	0.15	0.20	0.19	0.18	0.15	0.16	0.17	0.21	0.16	0.22

Source: Computed from WHO [103,104].

Note: Afghanistan (Afg), Algeria (Alg), Bahamas (Bah), Ecuador (Ecu), Ethiopia (Eth), Jordan (Jor), Lesotho (Les), Liberia (Lib), Marshall Islands (Mar), Bangladesh (Ban), Sudan (Sud), Uganda (Uga), Indonesia (Indo), Ghana (Gha), Mexico (Mex), South Africa (SA), India (Ind). Accuracy (ACC); true positive (TP or sensitivity); true negative (TN or specificity); false positive (FP); false negative (FN).

## 4 Discussion

To find the most reliable indices for predicting diabetes in low- and middle-income countries (LMICs), the study assessed fifty-four (54) biomarkers (22) and anthropometric (32) predictors of diabetes in adults in seventeen (17) LMICs. The variability observed in the predictive performance of biomarkers and anthropometric indices underscores the complex nature of diabetes and its underlying mechanisms in LMICs. It suggests that the risk factors and pathways leading to diabetes can differ among LMICs due to diverse genetic, health, environmental, and lifestyle factors [[54], [55], [56],^65^,^66^,^75^,^79^]. This finding emphasises the importance of considering country-specific factors and tailoring diabetes prevention and management strategies accordingly. Furthermore, diabetes remains a significant public health challenge among adults in LMICs and my study affirms the need for context-specific and objective diabetes screening tools to overcome the inadequacies of existing metrics [[105], [106], [107]].

The distribution of diabetes across LMICs shows notable divergence, with higher prevalence observed in certain regions. Specifically, Latin America, Asia-Pacific, and Middle East countries exhibited a higher burden of diabetes compared to other LMICs. For example, countries such as Mexico, Marshall Islands, India, and Jordan demonstrated a prevalence of diabetes that was more than ten times higher than that of Ethiopia and Liberia. This finding corroborates the studies by Vos et al. [56], Karter et al. [57], Luhar et al. [58], and Siddiqui & Donato [59], which highlight the doubling of diabetes prevalence in Jordan (69.7%), the impact of the obesity epidemic in the Marshall Islands on the rise of diabetes, and India's ranking next to China in terms of the diabetes epidemic. These findings underscore the substantial variation in diabetes burden among LMICs. The higher prevalence of diabetes in Latin America, Asia-Pacific, and Middle East countries may be attributed to a combination of factors. These regions often experience rapid urbanisation, sedentary lifestyles, shifts in dietary patterns, and an aging population, which are all known contributors to the increasing prevalence of diabetes. Additionally, genetic predisposition and differences in healthcare access and quality may also play a role in the observed disparities. Because diabetes remains a significant adult health challenge and the loss of diabetes care begins at the diagnosis stage [3,[108], [109]], efforts to improve the diagnosis of diabetes and risk factors such as obesity and hyperglycaemia should be considered.

Marshall Islands, Bahamas, and Mexico are the top three most obese countries [57,110] in this study. The distribution of obesity across different countries has been extensively studied, and various research studies have shown rapid adult obesity growth in LMICs. In studies conducted by Karter et al. [57], and Abarca-Gomez [111], it was reported that the Marshall Islands have experienced an obesity epidemic, which has contributed to the rise in diabetes prevalence in that region. The high prevalence of obesity in the Marshall Islands has been attributed to factors such as changing dietary patterns, sedentary lifestyles, and limited access to healthy food options. Similarly, Astudillo et al. [110], and Abarca-Gomez [111], also identified the Bahamas and Mexico as countries with significant obesity rates. These findings align with the global trend of increasing obesity rates, especially in LMICs. The rise in obesity has been linked to various factors, including urbanisation, changes in dietary habits, decreased physical activity, and socio-economic factors. The link between obesity and diabetes is well-established, with obesity being a major risk factor for the development of diabetes in adults.

Accordingly, the efficacy of BMI as a predictor of diabetes outcomes in these countries could potentially be attributed to the elevated prevalence of obesity in those regions. Furthermore, studies [58,59,73], have underscored the genetic predisposition of individuals of Asian, Hispanic, and African descent towards the accumulation of adipose tissue in the abdominal region, which is associated with unfavourable health outcomes [112]. And, in consequence, elevate central obesity, a salient driver of high blood sugar [113] and fat [114]. Therefore, the consistency of FPG, WHtR, ABSI, BF%, BRI, BAI, BPs, and BPd as reliable predictors of diabetes in LMICs may be due to the relationship between phenotype and diabetes. Also, multiple central obesity indices were stronger predictors of diabetes. Consistent with my results is research in HICs informing the prediction of diabetes with several anthropometric indices and biomarkers [4,5,34,37,39]. These results heighten the need for health and nutritional policies reform to include central obesity measures such as WHtR, ABSI, BAI, BF% and glucose indices like FPG as primary screening tools of diabetes in LMICs.

The finding that the prediction potency of other metrics outpaced BMI reaffirms observations made by several researchers in HICs and Asia [[115], [116], [117], [118], [119]] who reported BMI as a weak predictor of diseases including diabetes. It could be speculated that the inability of BMI to directly measure fat mass [119], differentiate between lean and fat mass and consider the effects of ageing and sex on adiposity and central obesity [120,121] elucidate the deficient performance. Contrarily, the more accurate diabetes prediction by WHtR, BF% and ABSI may be due to their direct measurement of fat mass and consideration of sex and age [122]. Undeniably, the utility of BMI is often due to convenience and cost [123,124] than reliability. Also, Haghighatdoost et al. [125], revealed that BMI might be a population-specific than a universal predictor of diabetes, a possible reason for prediction outcomes in the Marshall Islands, where more than 52% of the population are obese [57]. However, Fan et al. [126], study in China revealed that the reliance on low-cost and convenient disease markers led to poor disease detection. Similarly, reliance on weak disease markers negatively impacts disease misdetection [[105], [106], [107]]. Under this pretext, low-cost, effortless and reliable central adiposity indices for screening diabetes will be instrumental BMI surrogates in LMICs.

The findings highlight the superiority of FPG or FBG as the most potent predictor of diabetes, surpassing the predictive capability of BMI. Of relevance to this revelation are reports [113,[127], [128], [129], [130], [131]] who revealed better diabetes prediction outcomes with FPG than other metrics. Furthermore, research shows that higher blood glucose drives diabetes risk than other metrics across all ages, sex and ethnicities [132,133]. This underscores the critical role of FPG in capturing the true extent of an individual's diabetes risk. While physical adipose metrics, such as BMI, generally serve as indicators of diabetes risk [12,134] FPG is a confirmatory plasma venous determinant of diabetes [[135], [136], [137]] that is, FPG, arguably, perform biopsies, a valid and accurate diabetes diagnostic procedure [138]. Considering the dominant role of FPG among the available biomarkers, it becomes imperative to incorporate it as a mainstream index for disease screening. Furthermore, the utilisation of FPG as a primary screening tool can help overcome the challenges associated with population health screening in LMICs. Its simplicity and cost-effectiveness make FPG a viable option for widespread implementation in resource-constrained settings. By prioritising FPG as a key indicator for diabetes screening, LMICs can effectively allocate healthcare resources, develop targeted interventions, and mitigate the burden of diabetes within their populations. Similarly, the triglyceride glucose (TyG) index, a derived FPG index, has emerged as a promising screening tool for diabetes. The TyG index provides an integrated indicator of insulin resistance and metabolic dysfunction. This index offers advantages in terms of simplicity, as it utilises routine blood tests already conducted in clinical settings [139]. Through TyG index, healthcare providers in LMICs can assess an individual's risk of diabetes and potentially identify those who require further diagnostic testing or lifestyle interventions. Furthermore, the TyG index has shown relevance across different populations and ethnicities. Its consistent association with diabetes risk makes it a valuable screening tool for LMICs, where diverse populations with varying genetic backgrounds are present [[140], [141], [142], [143]]. Implementing the TyG index in diabetes screening can provide a standardised approach to risk assessment, ensuring consistency and comparability across different regions. By doing so, we can effectively monitor diabetes and address the challenges associated with population health screening in LMICs. This proactive approach would not only facilitate early detection of diabetes but also contribute to the mitigation of diabetes-related complications and the overall improvement of public health in LMICs.

The poor performance of the Bahamian, Ghanaian, South African, Mexican, and Indian models compared to Ethiopian, Ugandan, Jordanian, Sudanese and Basotho models could be due to reliance on anthropometric indices as the main indices for analysis of diabetes in the former group of countries. Studies including Al-Daghri et al. [144], and Criminisi et al. [145], reported high sensitivity outcomes for disease prediction models incorporating biomarkers and anthropometric indices. This reinforces the need for at least two responses, the first, thrusting the use of metrics other than BMI; the second, the simultaneous use of biomarkers and anthropometric indices than a single health metric for health measurements; the third, formulation of new indices and reformation of old indices especially BMI to incorporate age and sex while diagnosing or detecting diseases in LMICs.

### 4.1 Strength and limitations

This is the first study to provide a broad examination of biomarkers, anthropometric indices, and diabetes using multi-country surveys in LMICs. To this end, the study's findings can serve as the benchmark for designing, developing, and implementing better diabetes screening tools in LMICs. Moreover, the the findings of the study were explained in the context of regional characteristics, such as dietary patterns, genetics, healthcare access, and socio-economic factors to highlight why certain anthropometric measures or biomarkers were more predictive in specific regions. Despite the solidity of this text, the study has limitations. My study provides no longitudinal or age cohort analytical outcomes. In addition, the definition of diabetes was limited to self-reports with no single glucose or capillary glucose diabetes measurement in all surveys. These self-reports may either be overestimated or underestimate the true prevalence of diabetes. Also, in this cross-sectional study, the relationship between indicators is described by correlation and not causation though provide strong evidence for a positive association between glucose and diabetes. Moreover, the study's generalisability is limited due to the focus on LMICs and the inclusion of data from only 17 countries. The findings may not be representative of all LMICs or other regions not included in the study. Additionally, variations in healthcare systems, cultural practices, and socioeconomic conditions among the selected countries could affect the applicability of the findings to a broader population. Furthermore, the study's conclusions may not capture the full diversity and complexity of health-related issues in LMICs, as they are influenced by numerous contextual factors beyond the scope of the 17 countries studied. The study did not differentiate between diabetes type 1 and type 2, and the term "diabetes" was used in a general sense without specifying the specific forms. This decision was influenced by the lack of clarity in the data. Abdominal height was not directly included in the study's data. However, waist circumference (WC), which was included as one of the anthropometric indices, could serve as a surrogate or proxy for assessing abdominal adiposity. However, future research should focus on distinguishing between type 1 and type 2 diabetes and investigating the aetiological factors and measurements that can establish a cause-and-effect relationship between biomarkers, anthropometric indices, and diabetes across all LMICs.

## 5 Conclusion

Diabetes is burdensome in LMICs. Against a background of truncated diagnosis rates, inviting diagnosis and prediction metrics with more substantial accuracy is necessitated. In this study of 17 LMICs, I show variability in the association between biomarkers, anthropometric indices, and diabetes and suggested WHtR, ABSI, BF%, BRI, FPG and TyG for predicting diabetes. These findings and suggestions have a least four direct implications. The provision of the most relevant and current information on the nexus between biomarkers, anthropometric indices, and diabetes underscores the importance of context-specific diabetes detection indices. Furthermore, with such information clinicians or physicians will use evidence-based and context-specific indices for disease assessment to infer the presence or absence of disease, accurately confirm their diagnosis and suggest the best therapeutic options. Also, information on the evidence-based and context-specific will enhance public awareness of the dangers associated with malign health transpositions lifestyles. Moreover, this information can guide policymakers in implementing context-specific geriatric health and nutrition interventions and programmes in LMICs.

## Ethics approval and consent to participate

Not Applicable.

## Author contribution statement

1 - Conceived and designed the experiments.

2 - Performed the experiments.

3 - Analyzed and interpreted the data.

4 - Contributed reagents, materials, analysis tools or data.

5 - Wrote the paper.

## Data availability statement

Data associated with this study has been deposited at https://extranet.who.int/ncdsmicrodata/index.php/access_licensed/track/1391?sid=491;https://www.rand.org/well-being/social-and-behavioral-policy/data/FLS/IFLS/ifls5.html;https://www.iipsindia.ac.in/content/LASI-data.

## Funding

This work was supported by the 10.13039/100011326London School of Economics and Political Science (LSE) Postgraduate Studies Fund.

## Declaration of competing interest

The authors declare that they have no known competing financial interests or personal relationships that could have appeared to influence the work reported in this paper.

## List Abbreviations

A1C_DBS: Glycated Haemoglobin Dried Blood Spot
A1C-REV: Glycated Haemoglobin Reversal
ABSI: Body Shape Index
AC: Atherogenic Coefficient
Arm C: Arm Circumference
ADA: American Diabetes Association
AIP: Atherogenic Index of Plasma
AVI: Adiposity Volume Index
BAI: Body Adiposity Index
BP: Blood Pressure
BF%: Body Fat Percentage
BI: Broca Index
BMI: Body Mass Index
BRI: Body Roundness Index
CI: Conicity Index
CR: Cholesterol Ratio
CRR: Cardiac Risk Ratio
CR1I: Castelli's Risk Index-I
CR1II: Castelli's Risk Index-II
CRP-P-EQU: C-Reactive Protein Plasma Equivalent
CRP-DBS: C-Reactive Protein in Dried Blood Spot
FFM: Fat Free Mass
FFMI: Fat Free Mass index
FPG: Fasting Plasma Glucose
HAART: Highly Active Antiretroviral Therapy
Hb: Haemoglobin
HbA1c: Glycated Haemoglobin
HC: Hip Circumference
HDL: High-Density Lipoprotein
HI: Hip Index
HICs: High Income Countries
IDF: International Diabetes Federation
KH: Knee Height
LAP: Lipid Accumulation Product
LBM: Lean Body Mass
LDL: Low-Density Lipoprotein
LMICs: Low- and Middle-Income Countries
MENA: Middle East and North Africa
PIorRI: Ponderal Index or Corpulence Index or Rohrer's Index
SADC: Southern African Development Community Countries
SSA: Sub-Sahara Africa
STEPS: WHO Stepwise Approach to Non-Communicable Disease (NCD) Risk Factor Surveillance
RFM: Relative Fat Mass
RPI: Reciprocal Ponderal Index
TC: Total Cholesterol
TG: Triglycerides
THR: Triglycerides High Density Lipo-protein Ratio
TyG: Triglyceride-Glucose Index or Glycated Triglycerides
UAL: Upper Arm Length
UC: Urine Creatinine
UNICEF: United Nations Children's Fund
US: Urine Sodium
USAIDS: United States Agency for International Development
VAI: Visceral Adiposity Index
WC: Waist Circumference
WHO: World Health Organisation
WHtR: Waist to Height Ratio
WHT.5R: Waist to Height0.5 Ratio
WHR: Waist to Hip Ratio
WWI: Weight-adjusted Waist Index.
